# Efficacy and safety in enucleation of the prostate with thulium fiber laser (TFL) using a 365 μm fiber: a retrospective study in a real-world, risk-diverse population

**DOI:** 10.1007/s00345-025-05854-4

**Published:** 2025-08-13

**Authors:** Thomas Amiel, Ricarda Simon, Michael Straub

**Affiliations:** Department of Urology, TUM University Hospital Klinikum Rechts der Isar, TUM School of Medicine and Health, Ismaninger Strasse 22, 81675 Munich, Germany

**Keywords:** Benign prostatic hyperplasia, Endoscopic enucleation of the prostate, TFL, Fiber size, Anticoagulants

## Abstract

**Purpose:**

To assess the feasibility of using a 365 μm fiber for Thulium Fiber Laser Enucleation of the Prostate (ThuFLEP) in terms of settings, baseline characteristics, patient’s peri-operative outcomes and surgeon’s feelings.

**Methods:**

Retrospective single-center analysis of benign prostatic hyperplasia (BPH) patients that underwent ThuFLEP using a 365 μm fiber over an observation period of 14 months.

**Results:**

Analysis included 123 patients with mean age 72.4 years old (± 8.9) and average prostate size 94.8 cc (± 51.1); 34.1% of patients were undergoing antiplatelet or anticoagulant treatment before surgical intervention, and 50% maintained their antithrombotic during surgery. All had en-bloc enucleation of the prostate, with mean operation time of 115 min (± 41) and average resected tissue of 67.9 g (± 43). After the procedure, average catheterization time and hospital length of stay were 3 (± 0.9) and 3.6 days (± 1.4), respectively. Early post-operative complications occurred in 23 patients (18.7%). Twenty patients (16.3%) were readmitted to the hospital for check-up (6.5%) or emergency (9.8%), mainly due to catheter removal or macrohematuria respectively. Re-admission rate after one month was 4.9% (*n* = 6), mainly due to macrohematuria. The surgeon described a very precise and more focused laser beam compared to the 550 μm fiber with excellent instant coagulation during the enucleation.

**Conclusion:**

ThuFLEP using a 365 μm fiber allowed safe and effective enucleation of the prostate regardless of prostate size. While over a third of patients were under antithrombotic/anticoagulant treatment, half underwent surgery on continued medication without a significant increase of risks.

**Supplementary Information:**

The online version contains supplementary material available at 10.1007/s00345-025-05854-4.

## Introduction

Benign prostatic hyperplasia (BPH) surgical treatment relies on different techniques such as the “golden standard” transurethral resection of the prostate (TURP), open prostatectomy, and anatomical endoscopic enucleation of the prostate (AEEP) using lasers [1–3]. The Thulium Fiber Laser (TFL) has recently emerged as an efficient therapeutic option to treat BPH with promising clinical and safety outcomes [2]. TFL wavelength of 1940 nm with its pulsed mode provides very low penetration depth leading to precise tissue cutting with minimal carbonization effect thanks to heat dissipation between laser pulses [3]. The continuous-wave mode provides maximal hemostasis and coagulation, comparable to what can be seen with the Thulium: YAG laser [4, 5].

However, optimal laser settings and fiber sizes are still poorly determined. According to experts in laser use, the most frequently used fiber for prostate enucleation is 550 μm; standard settings for TFL are 1.5 J ( 1.0–1.5 J), 35 Hz (15–60 Hz), and 60 W (30–60 W) for apical and main gland AEEP, and 1 J (1–2 J), 30 Hz (20–40 Hz) and 30 W (20–60 W) for coagulation [6]. Functional outcomes and safety parameters of the TFL have been assessed with the 550 μm, 600 μm fibers, occasionally the 800 μm fiber [7, 8] and the 1000 μm fiber, but not with the 365 μm fiber. However, they may be of interest since they deliver a beam with higher density of energy, expected to be associated with higher precision in cutting. The use of the smaller fiber allows a more precise knife-like cutting through the tissue of the prostate without burning the parenchyma. The aim of this single-center retrospective study is to assess the feasibility of Thulium Fiber Laser Enucleation of the Prostate (ThuFLEP) using a 365 μm fiber and to report the initial experience including surgeons’ feelings, TFL settings, patient’s profile, and complications within the first 30 days after surgery in a real-life population presenting with various risks.

## Methods

Following institutional ethics approval (2024-572-S-NP), a retrospective, single-center analysis was performed of all BPH patients at our institution MRI-Endourology, University Hospital Klinikum rechts der Isar, Technical University Munich, who underwent a ThuFLEP with a 365 μm fiber from September 2022 to December 2023 by a single surgeon.

### Inclusion criteria

This study collected patients treated for the first time for BPH and not diagnosed for prostate cancer or hematological disease prior to the procedure. There were no restrictions for age, anticoagulant treatment, or prostate size.

### Surgical technique

Surgeries were carried out using the TFL-Drive (Coloplast, Denmark) equipped with presettings for the 550 μm-fiber or with another Quanta system TFL together with the 26 French continuous flow laser resectoscope (Richard Wolf, USA) equipped with a 30° telescope optics. All surgeries were en-bloc enucleations and used a 365-µm fiber with following settings: 1.2 J, 30 Hz (i.e. 36 W), short pulse for enucleation and 0.6 J, 40 Hz (i.e. 24 W) long pulse for coagulation. For morcellation of the enucleated adenoma the Richard Wolf Piranha morcellator was used.

### Statistical analysis

Statistical analysis is descriptive. For quantitative variables, mean, standard deviation (SD), median, range (min, max) and interquartile range (IQR) were calculated; qualitative variables are expressed in numbers and percentages.

### Outcomes

Pre-operative data, hospital length of stay (LOS), resected prostate volume, surgical complications according to Clavien-Dindo scale, reoperation, and early and late patient readmission (after 30 days) were assessed. Enucleation and morcellation times were not recorded, so surgery efficiency was assessed through operative time and total procedure efficiency. Surgeon’s feedback analysis was analyzed from surgery reports and surgeon’s cumulative experience.

## Results

### Baselines characteristics

The study cohort included 123 patients (Table 1) with mean age of 72.4 years old ± 8.9 (53–95 y; median: 73 y) and average prostate size of 94.8 g ± 51.1 (27–300 g; median: 85 g). 52 patients (42.3%) had a preoperative indwelling catheter. 42 patients (34.1%) were under ongoing antithrombotic treatment before the surgical intervention, either antiplatelet (AP) or anticoagulant (AC): 18 under acetylsalicylic acid (ASS), 21 under anti-Xa drugs and 3 under vitamin K antagonist (VKA). During the surgery half of them (*n* = 21) maintained their treatment. After ThuFLEP, 29 patients (23.6%) were diagnosed with prostate cancer (PCa).

### Operative outcomes

The mean operation time was 115 min ± 41 (59–258 min) with an average resected tissue volume of 67.9 g ± 43 (10–250 g) and total procedure efficiency (from introduction of the endoscope until the placement of the catheter) of 0.57 g/min ± 0.26 (0.11–1.2 g/min). A slight improvement in efficiency is observed when patients are divided into two groups based on the time at which ThuFLEP was performed. For the cohort of the first half (September 2022 to mid-July 2023), the mean efficiency was 0.53 g/min (62 patients, mean prostate size 88.7 g), while for the cohort of the second half (mid-July 2023 to December 2023), the mean efficiency was 0.61 g/min (61 patients, mean prostate size 101.1 g). When splitting our cohort in two subgroups based on prostate size, an average efficiency of 0.41 g/min for prostates ≤ 80 g (*n* = 57), compared to 0.70 g/min for prostates > 80 g (*n* = 66) was observed.

Average catheterization time after the procedure was 3 days ± 0.9 (2–8 days), almost fitting the average hospital LOS (3.6 days ± 1.4; 2–8 days).

### Peri-operative complications

Peri-operative complications (Table 2) were observed in 23 patients (18.7%), resulting in 9 patients (7.3%) with grade I, 1 patient (0.8%) with grade II and 13 patients (10.6%) with grade III (grade IIIa (*n* = 2) and grade IIIb (*n* = 11)) according to Clavien-Dindo classification. The most frequent complications were bleeding (*n* = 9) and urinary retention (*n* = 5). Out of the 9 patients with bleeding, 5 patients (11.9%) were under daily anti-thrombotic treatment from which 1 patient who continued treatment during surgery and 4 patients who stopped it 48 h to 72 h prior to the surgery. Regarding urinary retention, out of the 5 patients, 3 patients were under ongoing AP therapy during surgery and 1 patient paused anti-Xa 48 h before procedure.

Reoperation, mainly due to macrohematuria, was performed in 13 patients (10.6%) with one procedure within 7 days after the initial ThuFLEP. All these patients were in the grade III Clavien-Dindo complications group; 8 of them belonged to the group of patients under AC or AP, of which 4 were under ongoing treatment at the time of the surgery and 8 patients presented large prostate volumes above 85 g.

After discharge from the hospital, 20 patients (16.3%) experienced an early re-admission, either for check-up (6.5%, *n* = 8) or for emergency (9.8%, *n* = 12). The reason for check-up was mainly due to catheter removal (*n* = 3). Patients with emergency re-admission were primarily treated for macrohematuria (1 under anti-Xa, 1 under AP, and 1 under VKA treatment maintained during surgery, while 1 more paused AP treatment 48 h before procedure) and post-operative cystitis (UTI) (4 patients, of which 3 with prostates > 100 g). The re-admission rate after one month was 4.9% (*n* = 6), mainly due to macrohematuria (*n* = 5). Of the readmitted patients, 4 had continued AP or AC during surgery, and 1 had paused anti-Xa drug prior to surgery.

The complication rate within the subgroup of patients diagnosed with incidental PCa (Supplementary Table 1) was 17.2% (*n* = 5).

### Surgeon’s feedback

The surgeon described excellent dissection along the surgical planes during the enucleation while allowing very high precision without losing good visibility and maintaining excellent hemostatic properties.

**Table 1:** Baseline characteristics and patients’ operative outcomes

Baseline characteristics	ThuFLEP (*n* = 123)
Age (years) Mean ± SD (range, median, IQR)	72.4 ± 8.9 (53–95, 73, 13)
Prostate volume (g) Mean ± SD (range, median, IQR)	94.8 ± 51.1 (27–300, 85, 57.5)
Indwelling catheterization before surgery	52 (42.3%)
Patients under antithrombotic treatment	42 (34.1%)
Anti-Xa (apixaban, endoxaban, rivaroxaban)	21 (17.1%)
Acetylsalicylic acid (ASS)	18 (14.6%)
VKA (Vitamin K antagonist: phenprocoumon)	3 (2.4%)
Patients under antithrombotic treatment maintained during surgery	21 (17%) (anti-Xa (4), ASS (16), VKA (1))
Patients under antithrombotic treatment paused for the surgery	21 (17%) (anti-Xa (17), AVK (2), ASS *n* = 2)
Patients under a second antithrombotic treatment (maintained during surgery)	2 (1.6%) (clopidogrel (1), ASS (2))
Incidental pathology	
Prostate cancer (PCa)	29 (23.6%)
Lymphoma	1 (0.8%)
Operative outcomes	
Operative time (minutes) Mean ± SD (range, median, IQR)	115 ± 41 (59–258, 105, 44)
Resected tissue volume (g) Mean ± SD (range, median, IQR)	67.9 ± 43 (10–250, 65, 51.5)
Total efficiency procedure* Mean ± SD (range, median, IQR) (g/min)	0.57 ± 0.26 (0.11–1.2, 0.55, 0.41)
First half patients treated (*n* = 62) *mean*	0.53 g/min
Second half patients treated (*n* = 61) *mean*	0.61 g/min
Prostate sizes ≤ 80 g (*n* = 57) *mean*	0.41 g/min
Prostate sizes > 80 g (*n* = 66) *mean*	0.70 g/min
Catheterization time (days) Mean ± SD (range, median, IQR)	3 ± 0.9 (2–8, 3, 1)
Hospitalization time (days) Mean ± SD (range, median, IQR)	3.6 ± 1.4 (2–8, 3, 1)

*From introduction of the endoscope until the placement of the catheter. Ranges indicate the minimum and maximum values

**Table 2:** Peri-operative outcomes and patients’ readmission

			Patients under antithrombotic treatment	
	Overall (*n* = 123)	Patients without antithrombotic treatment (*n* = 81)	Antithrombotic maintained during surgery (*n* = 21)	Antithrombotic paused before surgery (*n* = 21)
Clavien-Dindo complications*	23 (18.7%)	9 (11.1%)	8 (38.1%) (ASS (5), anti-Xa (2), VKA (1))	6 (28.6%) (anti-Xa (5), VKA (1))
Class I	9 (7.3%)	4 (4.9%)	3 (14.3%) (ASS)	2 (9.5%) (anti-Xa)
Urinary retention	5 (4.1%)	1 (1.2%)	3 (14.3%) (ASS)	1 (4.8%) (anti-Xa)
Macrohematuria	3 (2.4%)	3 (3.7%)	-	-
Both bleeding and urinary retention	1 (0.8%)	-	-	1 (4.8%) (anti-Xa)
Class II**				
Infection	1 (0.8%)	-	1 (4.8%) (anti-Xa)	-
Class III	13 (10.6%)	5 (6.2%)	4 (19.0%) (ASS (2), anti-Xa (1), VKA (1))	4 (19%) (anti-Xa (3), VKA (1))
Class IIIa	2 (1.6%)	1 (1.2%)	1 (4.8%) (ASS)	-
Urinary retention	1 (0.8%)	1 (1.2%)	-	-
Kidney ectasia	1 (0.8%)	-	1 (4.8%) (ASS)	-
Class IIIb	11 (8.9%)	4 (4.9%)	3 (14.3%) (ASS (1), anti-Xa (1), VKA (1))	4 (19%) (anti-Xa (3), VKA (1))
Bleeding requiring re-intervention**	9 (7.3%)	4 (4.9%)	1 (4.8%) (anti-Xa)	4 (19%) (anti-Xa (3), VKA (1))
Urinary retention	2 (1.6%)	-	2 (9.5%) (VKA (1), ASS (1))	-
Reoperation (within 7 days after ThuFLEP)	13 (10.6%)	5 (6.2%)	4 (19.0%) (ASS (2), anti-Xa (1), VKA (1))	4 (19%) (anti-Xa (3), VKA (1))
Early re-admission (7 days after surgery)	20 (16.3%)	11 (13.6%)	5 (23.8%) (anti-Xa (2), ASS (2), VKA (1))	4 (19%) (anti-Xa (3), ASS (1))
Check-up***	8 (6.5%)	4 (4.9%)	2 (9.5%) (anti-Xa (1), ASS (1))	2 (9.5%) (anti-Xa)
Catheter removal	3 (2.4%)	2 (2.5%)	-	1 (4.8%) (anti-Xa)
UTI	2 (1.6%)	1 (1.2%)	1 (4.8%) (anti-Xa)	-
Macrohematuria	1 (0.8%)	-	-	1 (4.8%) (anti-Xa)
Nephrostomy	1 (0.8%)	-	1 (4.8%) (ASS)	-
Scrotal edema	1 (0.8%)	1 (1.2%)	-	-
Emergency	12 (9.8%)	7 (8.6%)	3 (14.3%) (anti-Xa (1), ASS (1), VKA (1))	2 (9.5%) (anti-Xa (1), ASS (1))
Macrohematuria	5 (4.1%)	1 (1.2%)	3 (14.3%) (anti-Xa (1), VKA (1), ASS (1))	1 (4.8%) (ASS)
UTI	4 (3.3%)	4 (4.9%)	-	-
Urinary retention	2 (1.6%)	1 (1.2%)	-	1 (4.8%) (anti-Xa)
Balanitis	1 (0.8%)	1 (1.2%)	-	-
One-month re-admission	6 (4.9%)	1 (1.2%)	4 (19.0%) (ASS (2), anti-Xa (1), VKA (1))	1 (4.8%) (anti-Xa)
Macrohematuria	5 (4.1%)	1 (1.2%)	3 (14.3%) (anti-Xa (1), VKA (1), ASS (1))	1 (4.8%) (anti-Xa)
Bleeding	1 (0.8%)	-	1 (4.8%) (ASS)	-

*UTI* Urinary tract infection, *ASS* acetylsalicylic acid, *VKA* Vitamin K antagonist

*Only 1 patient reported 2 types of Clavien-Dindo complications (bleeding and urinary retention) while the rest only presented 1 type of Clavien-Dindo complication

**2 patients of Clavien Dindo 3 complications received a blood transfusion (Clavien Dindo II), one with antithrombotic therapy maintained during surgery and one with paused antithrombotic therapy before surgery. As transfusions were also associated with reintervention, they are presented only in Clavien Dindo III group

***This clinical control was already planned and not a spontaneous check-up

## Discussion

This retrospective single-center study provides initial information on the outcome of TFL AEEP with a thinner fiber than previously used. The analysis includes general surgical parameters, perioperative and short-term complication data and surgeon feedback on feasibility.

Within this analysis, the mean age of patients was 72.4 years old, slightly older than in other studies using TFL, where mean age was between 66 and 69-years old [3, 8–15]. Sixty-four patients had small to medium prostate sizes (27–85 g) and 59 patients had large prostates (90–300 g) with 15 being over 150 g. Regardless of this size allocation, patients were treated successfully. A similar patient population was presented by Petov and colleagues with a mean prostate volume of 86.9 g (20–330 g), where ThuFLEP was performed using the 600 μm fiber. Authors concluded the procedure as an efficient alternative to other laser surgeries such as HoLEP [14]. While HoLEP has been shown to be efficient, safe and feasible procedure even in very large prostates (> 175 mL) [16], few data is currently available for ThuFLEP. However, a recent retrospective analysis concluded that ThuFLEP and HoLEP had similar efficacy and safety outcomes in patients with prostates larger than 175 ml [17]. Overall, our findings suggest a good feasibility of using the 365 μm fiber for ThuFLEP in patients with all prostate sizes, and of any age.

The standard catheterisation duration in our clinic was reduced from 2 to 3 days during the study period, so the reported catheterization time – close to that of other studies- should be interpreted with caution. Mahajan et al. reported in their study an average indwelling time of 2.7 days for ThuFLEP, having treated a younger population of mean 68.1 years old and smaller prostate range (82–90 cc) [13]. Similar results were found by Bozzini et al. with an average indwelling time of 2.5 and 2.6 days for Thulium: YAG and continuous-wave (CW) ThuFLEP respectively [7].

Mean LOS was 3.6 days, comparable to the results of Petov et al. of 3.7 days [14], but higher than those of Serezo et al. (1.8 days) [15]. in the subgroup of patients with more than 3 days LOS (55 patients) the average prostate size was 100.5 g-suggesting that longer hospitalization may be related to larger prostates- and 26 patients were under anti-Xa or AC (with 12 who maintained their treatment during surgery). Aligned with our findings, studies have shown that AC/AP patients treated for BPH with other technologies such as HoLEP, Thulium: YAG or Diode Laser Enucleation of the Prostate had a prolonged LOS with ranges between 4 and 8.7 days [18–20].

Early perioperative complications after ThuFLEP were experienced by 23 patients, representing 18.7% of the cohort, fitting usual ranges reported in the literature (15.5–22% [7, 8, 12, 15, 21] and up to 25–45% [3, 13, 14, 22]). According to Clavien Dindo classification, in our study 7.3% of patients faced grade I complications similar to the 6% rate observed by Enikeev et al. [21], lower compared to similar studies reporting 10–31% [7–9, 12–14], but higher than the 1.4% rate observed by Serezo et al. [15]. Only 1 patient faced grade II complication (0.8%), whereas other studies described rates of 2 to 14% [3, 7–9, 13–15, 21–23].

We observed 10.6% of patients (*n* = 13) in our series with grade III complications (bleeding the most common complication with 7.3%: 4.1% under antithrombotic treatment and 3.2% without), which is higher than the rates reported in by Enikeev, Morozov, and Petov (3.3, 2.0 and 1.9% respectively [14, 22, 23]). However, they might have excluded patients under AC/AP treatments, which would not reflect the real life, as a large part of the aging population is under AC/AP treatment [24, 25]. In Serezo et al. study [15], 30% of patients were under AC/AP (whether they stopped drugs before procedure is unknown) and the rate of grade III complications was still low (2.8%) while grade II rate was higher than ours (9.9% vs. 0.8%). It is worth reminding that patients under AC/AP treatment prior to prostate surgeries (HoLEP [18] /TURP and PVP surgeries [26]) have increased risk of postoperative bleeding.

In our cohort, the higher percentage of patients with early bleeding complications seems to be directly linked to the fact of AC or AP treatment (14.3%) when compared against patients without antithrombotic treatment (4.9%), with little influence of whether they had stopped or continued their treatment during the surgery. When combining the bleeding complications during surgery with those occurring in the 30-day period after the surgery, no clear difference between the patients that had stopped or continued their AC/AP treatment during the surgery is observed.

Early readmission to the hospital is somewhat difficult to discuss as it is usually not reported in clinical studies. Safety and efficacy are reflected in the low incidence identified for late readmission at the hospital (after 30 days) with 6 patients (4.9%) mainly with macrohematuria. Morsy and colleagues analyzed complications after 1 month and observed a rate of 17.9% in patients that underwent low-power ThuLEP, mainly reporting urge and stress urinary incontinence [27]. Castellani and associates identified comparable rates to ours with 3% complications in their patients after ThuFLEP [9]. Regarding a medium-term follow-up of 3 months after ThuFLEP (not assessed in our study), two other studies reported 3% and 3.5% rates of patients facing complications grade I mainly due to stress urinary incontinence [21, 23].

In line with other cohorts after HoLEP [28], 29 patients (23.6%) were diagnosed with PCa after ThuFLEP. Among them, only 5 experienced early postoperative complications. Similar to our results, Marchioni et al. found that TURP can be performed safely and efficiently in patients with incidental PCa, with no significant difference in outcomes between PCa and non-PCa patients (similar hospital length of stay, complications, readmission and mortality rates [29]). Becker and associates report that HoLEP is feasible, safe and effective, in patients known to have a PCa, reporting a 20.1% overall rate of complications comparable to our series [30]. Overall, our results suggest that ThuFLEP with 365 μm fiber is a feasible and safe surgery for BPH treatment in patients with PCa.

The retrospective and the non-comparative design of this study are the main limitations. Thus, a prospective study comparing different fiber sizes could assess if one fiber size is more convenient to another for efficiency (including enucleation efficiency, for which data were not available in the current study) and safety outcomes, as well as for surgeon’s comfort. Moreover, longer patient follow-ups would allow us to compare our findings to other studies in the literature and to confirm the safety and retreatment rates of the procedure.

Despite these limitations, this study is the first to report the efficacy and safety outcomes of ThuFLEP using a 365 μm fiber with widely open inclusion criteria (no exclusion of patients with big prostates, or with AC/AP treatments), fitting with real life data. This gives strength to the findings and provides data on feasibility and safety for BPH treatment on a high-risk population.

Otherwise, manufacturers should be encouraged to define presettings also for the 365 μm-fiber.

## Conclusions

ThuFLEP using a 365 μm fiber allowed safe and effective enucleation of the prostate regardless of prostate size in a patient cohort with a higher mean age than usually reported and with more than 30% of patients under antithrombotic treatment, half of whom maintained their medication during surgery. The surgeon described excellent dissection along the surgical planes allowing very high precision without losing good visibility and maintaining excellent hemostatic properties. The most frequent early surgical complications were bleeding and urinary retention. Early and late readmission rates were in a similar range as reported in the literature. Further randomized prospective studies are needed to confirm these initial results.

## Supplementary Information

Below is the link to the electronic supplementary material.

Supplementary file1(DOCX 22 KB)

## Data Availability

No datasets were generated or analysed during the current study.
