# Supplementary material for: Efficacy and safety in enucleation of the prostate with thulium fiber laser (TFL) using a 365 μm fiber: a retrospective study in a real-world, risk-diverse population
Source: World J Urol. 2025 Aug 13;43(1):491. doi: 10.1007/s00345-025-05854-4 (PMC12350578; doi:10.1007/s00345-025-05854-4)
Supplement: Supplementary file 1 — Supplementary file1(DOCX 22 KB) [file 345_2025_5854_MOESM1_ESM.docx]

**Supplementary table 1. Clavien-Dindo complications in incidental PCa patients**

|  |  |  | Patients under antithrombotic treatment | |
| --- | --- | --- | --- | --- |
|  | **Overall (n=123)** | **Patients non-AC/AP (n=81)** | **Maintained during surgery (n=21)** | **Paused before surgery (n=21)** |
| Incidental PCa patients’ complications |  |  |  |  |
| Class I | 2 (1.6%) | - | 1 (4.8%) (ASS) | 1 (4.8%) (anti-Xa) |
| Urinary retention | 1 (0.8%) | - | 1 (4.8%) (ASS) | - |
| Bleeding, urinary retention | 1 (0.8%) | - | - | 1 (4.8%) (anti-Xa) |
| Class IIIa – Urinary retention | 1 (0.8%) | 1 (1.2%) | - | - |
| Class IIIb | 2 (1.6%) | - | 1 (4.8%) (VKA) | 1 (4.8%) (anti-Xa) |
| Bleeding | 1 (0.8%) | - | - | 1 (4.8%) (anti-Xa) |
| Urinary retention | 1 (0.8%) | - | 1 (4.8%) (VKA) | - |

**PCa:** Prostate cancer, **AC:** anti-coagulant, **AP:** anti-platelet, **ASS:** acetylsalicylic acid, **VKA:** vitamin K antagonist
